# Supplementary material for: Comment on Dimou et al. Profile of Membrane Cargo Trafficking Proteins and Transporters Expressed under N Source Derepressing Conditions in Aspergillus nidulans. J. Fungi 2021, 7, 560
Source: J Fungi (Basel). 2021 Dec 3;7(12):1037. doi: 10.3390/jof7121037 (PMC8703528; doi:10.3390/jof7121037)
Supplement: Supplementary file 1 [file jof-07-01037-s001.zip › jof-1445904-supplementary.pdf]

>AFUA_5G05910/Afu5g05910 [Aspergillus fumigatus Af293] genomic sequence with new ATG and former **ATG**, wrongly predicted to be the start codon in AspGD and FungiDB. Non-canonical (GC-AG) intronic region, 110 nt.

ATGGCACCGGCTATTCCCTCGGCCAAGTTGACTCTGTCTTGTCCCCTCTTCGCGGCCGATTTCGATCCGC
GCAACC**ATG**GCTTCTTGCTCGTTGGAGGCGGTGGAGGTGAAGGACGTAGTGGCGTTGGAAATAAAATTGC
AAGTTCCCCTATACTCTACTACTAGGATCATGCGATCCGCACACATCGTGACGTTGATTCTTCTATACCA
AGGCATACGCTAATTTTGAGAGCCGACTTGGAACACAGGCGCTGTTGGACACTTCAAAACGCAACGAAAT
CTTGGAGGTAGTCGATATTGAACTTTCTCGCGATGAGGATTCCGTTACTTCGCTAGCCGCTGCGCAAGCG
AGTGATGAATCGGTCATTGCCCTGGCCGGTATCAACAGCTCCGTGGCCGAGCAGAAGAGGAACAACAATG
AACATTTGAGATCCTTCCAGATCGACTATCCCCCGCGGAAAAGAAGGTTGGCGAACACTTCCGGAGAAGA
AGCAAAGACAGTCAAAGAGAATCCCCGTCCAGGAAAGACTAGAGCACTAGCACGTGTGTCACTGTTTCGG
ACAAAGGAGTCCAAGGCATCCAAGGCTGGCTCCGATACCTATCAGAGGATATTGCGACTATCCCCATGGA
AAGATGGGGACGCTCCCAGAGTAGGTGCCATTGCGACCGGGCTAGCTCCTGCGGGGGAAATCGTATTCTT
CAAAGTCACTCCCACGCCGACCGAGGCTGATGTCATTGGGAGGATTCGCCTGAACAGTGAGGAGGAAGCT
GAGGATGTGGATATCATCGACCTGGAGGACGATAGGAAATACAAAGTGGCCTACACCAACGGGAATGACG
TTTTTATTTGTGAAATAACATTACAGACAAGGTCAAACACCGCTCCAGATGTGCAGTGCGTCTACAGCAC
ACCTCTGCCCGAAAAGGGACTGAGGACAAGGCCTAAGTTTCGAGCGCTGCGTTTCCTCTCCCCCACAACA
CTGTTGCTTCTCCAGAATGCACCAAACCGAAGCGGTTGCGAGCTTTTGATTCTAAATTTACGGCCGTTTT
CGAAAGATAAGCCTTCCGGGGTCATTGTAAGGCGCAAGAAGCTGCGCAAATCAATGAAGATCGGGCTAGG
TCTGGACACTTGTGGTTTAGGAACGAATCCAGATGGCCAGCAGCAGACCATTATTGCGGTATCTGGTAGC
GACCAGTCCATCGAGGTCTTGGCAGTTGAGTTCAGTGCAAGAAAGGGCTACGGCAAACTCCGGCCGTATA
CAACCCTACGAGACGTCCACCCGTTCTCGATGACCAAGATCTGCTTCTCAAACTTCATCCCCCCGGCGCA
TCCCGTCACGCCAGAGACCCCTCCTCAATATGTCAAGCTGGCCTCGATCAGCATGGGCAACACGGTTGTC
GTGCACACCTTCCCGCTCGCCCCATCACCGCCCTCCAGCCGCACCCCACGGTACGTGCTGGTCATGCCCG
GCGAGTCGGAGATCTGGACCAACTTCGCCAGCGGCTTTGCAGCCCTGCTCTCCATTATCACCGTCTGCAT
CCTCCTCCAGGCCTTCACGGAGATCAGAGGCCTCATGCCCCCGTACCTGGGGGCCTCCGCGTGGCTTCCC
CCGGATATCCGCGCGGCCATTGCGCGCCCGTACCACCCCCTCCCGCCTCACCCTCTCCCAACCGCATCCG
CGCTCTCGGCCCATACCGATTCCCCAAAGCAGACCCTCCGAGATCTCCTTCATGCCCGTCATGCCGCCGG
GGCCAACGATGCCGATGTGGCCCCGGACGCTGATGCAACGACTCCAGCTAACTCGGTCTTTGTCCGATGT
CTGCCTGAATCCAACAACATCTACATCGAGAGCTCTGACGCTCATTCCTCCCGCTCATCTTCGTCATCTC
CTGAAGAGGTAGAAGAGCAGCCATCCCGATCCTGGGATGACCTCGACGAGGAGGAGCGTTCTGCCTGGAA
GCAGCGCTTGATCGATGCTGGGTACTGGGCTCTCGAAGAGGGGGAGACGATCCTCCAAGGTGTATTGTTC
GGCGAATGCTCTGCCTTTCTGGGTAGTCAAGTGCAAGCTGAGCTTCAATCTTAG

AFUA_5G05910/Afu5g05910 [Aspergillus fumigatus Af293] 657 aa

'New' protein sequence considering the new ATG start codon.

MAPAIPSAKLTLSCPLFAADFDPRNHGFLLVGGGGGEGRSGVGNKIALLDTSKRNEILEVVDIELSRDED
SVTSLAAAQASDESVIALAGINSSVAEQKRNNNEHLRSFQIDYPPRKRRLANTSGEEAKTVKENPRPGKT
RALARVSLFRTKESKASNAGSDTYQRILRLSPWKDGDAPRVGAIATGLAPAGEIVFFKVTPTPTEADVIG
RIRLNSEEEAEDVDIIDLEDDRKYKVAYTNGNDVFICEITLQTRSNTAPDVQCVYSTPLPEKGLRTRPKF
RALRFLSPTTLLLLQNAPNRSGCELLILNLRPFSKDKPSGVIVRRKKLRKSMKIGLGLDTCGLGTNPDGQ
QQTIIAVSGSDQSIEVLAVEFSARKGYGKLRPYTTLRDVHPFSMTKICFSNFIPPAHPVTPETPPQYVKL
ASISMGNTVVVHTFPLAPSPPSSRTPRYVLVMPGESEIWTNFASGFAALLSIITVCILLQAFTEIRGLMP
PYLGASAWLPPDIRAAIARPYHPLPPHPLPTASALSAHTDSPKQTLRDLLHARHAAGANDADAAPDADAT
TPANSVFVRCLPESNNIYIESSDAHSSRSSSSSPEEVEEQPSRSWDDLDEEERSAWKQRLIDAGYWALEE
GETILQGVLFGECSAFLGSQVQAELQS

Figure S1: Amended gene structure and conceptual translation of the *A. fumigatus* strain Af293 AN11127/Sec12 orthologue
